# Veterinary Notes from Europe1An address delivered at the semi-annual meeting of the Pennsylvania State Veterinary Medical Association, Pittsburg, September 20, 1898.

**Published:** 1898-09

**Authors:** Leonard Pearson

**Affiliations:** Dean, University of Pennsylvania, Veterinary Department


### VETERINARY NOTES FROM EUROPE.[1]

By Leonard Pearson, B.S., V.M.D.,
DEAN, UNIVERSITY OF PENNSYLVANIA, VETERINARY DEPARTMENT.

During the past summer I have had an opportunity of attending the Congress for the Study of Tuberculosis that met in Paris, from July 27th to August 2d, and of making a hurried trip to a few of the capitals of Europe for the purpose of investigating the measures employed for combating tuberculosis of cattle. Incidentally, I have been able to pick up a number of points of general veterinary interest. In accordance with your invitation, extended through the President and Secretary, I take pleasure in informally presenting to you a few veterinary notes from Europe.

The Tuberculosis Congress was made up of about 250 delegates and members from the various countries of Europe and two from the United States. The meeting was held in the École de Medecine. The present Congress was the fourth of the series. The President for this year was Professor Nocard, of the National Veterinary School at Alfort. This no doubt encouraged a large veterinary attendance; many of the members, delegates, and officers were of the veterinary profession. During the progress of the meeting more than one hundred papers were read. It is not possible to review these papers, or even those that are of greatest importance, without occupying more time than can be devoted to this discussion. I will therefore call attention to a few points that impressed me as being of especial importance.

The first paper, by Professor Bang, was on "Prophylactic Measures Against Tuberculosis of Animals." In this paper the prophylaxis of tuberculosis in various parts of the world was discussed and the importance of some efficient measures of prophylaxis was emphasized. Then the methods employed in Belgium and Massachusetts were considered in great detail as examples of a system of suppressing this disease. In both States the attempt was made to buy up and destroy all tuberculous animals, making more or less systematic examinations of the cattle, and in both States, after an expenditure of considerable amounts and a trial extending over several years, the methods were given up as not feasible. Then the methods employed for the same purpose in Denmark were described in detail.

[1] An address delivered at the semi-annual meeting of the Pennsylvania State Veterinary Medical Association, Pittsburg, September 20, 1898.

Much has been said of the Danish method for suppressing tuberculosis of cattle, and there has been much interest in it in this country. Indeed, it has been warmly advocated in the United States as the true solution of the whole question of suppressing tuberculosis of cattle. In order that we may understand the Danish, or the Bang, system of dealing with tuberculosis, it is necessary for us to understand the agricultural conditions that prevail in Denmark. So that, if you will permit me, I will diverge for a moment and say a few words in reference to these conditions..

Denmark is a comparatively small country, having an area of but 15,000 square miles, or about one-third the area of Pennsylvania. It has, however, fully as many cattle and, perhaps, a few more than this State. Its cattle population is slightly above 2,000,000; it also has about the same number of people. Its one large city, Copenhagen, has a population of 600.000. The country is divided into two parts, the peninsula, or Jutland, and the island, or Seeland. The shores of both of these parts receive the balmy influence of the Gulf Stream, which tends to greatly moderate the severity of the climate that this northern country would otherwise have. It is interesting to note that Denmark is situated as far north as the Hudson Bay. The country is a gently rolling plain, sparsely wooded in parts, but generally open. It is a very good grass country and well adapted for dairying. It is not an exaggeration to say that Denmark is the dairy farm of England. Indeed, a very large proportion of the money that comes into the country is received from England for dairy products. Danish butter has an exceedingly high reputation on the European market. It is of excellent quality, and is uniform; it finds a ready sale not only in England, but also in France, and to a smaller extent in Germany, and it is exported to Hong Kong and Canton.

As a dependency on the dairy industry, the rearing and feeding of swine is gone into quite extensively, skim-milk being used largely for this purpose. The butter of Denmark is not made on the individual farms, but is made in coöperative creameries; a number of farmers in a district combine, build a creamery, send their milk to this central station, and have it manufactured by a skilled buttermaker. It is this system that is largely responsible for the uniformity and quality of Danish butter. It is the custom in Denmark for the cream, after it is separated, to be heated to about 80° C.; it is then ripened preparatory to churning.

The skim-milk is heated to a higher point, or is even boiled, before it is returned to the farm. These temperatures are high enough to destroy the tubercle bacilli, and milk thus treated can be used without danger.

Now that we have thus briefly reviewed the system of agriculture that is generally practised, let us consider the Bang method of dealing with tuberculosis of cattle. His method is to test with tuberculin, and then divide the herd under examination into two parts, or into two herds, one containing the cattle with regard to which there is no question, that are believed to be healthy; the other containing the animals that have responded to the tuberculin-test and those that are looked upon as suspicious. These herds are, if possible, quartered in separate buildings. If this is not possible, the cow-stable is divided into two distinct parts by means of a tight board partition, and, as a rule, there is no internal communication between the stables. It is necessary to go out-doors to pass from one to another. The two lots of cows are cared for by separate attendants, or if the herd is small and it is absolutely necessary for the same attendants to care for both lots they are obliged to have and to wear separate shoes and outer clothing when in the stable of the reacting section. The cattle that show physical signs of tuberculosis are slaughtered. The calves from the cows in the reacting sections are saved. These calves are fed on the milk of healthy cows or on the heated milk of reacting cows. The raw milk of reacting cows is never used for calf-feeding. The calves are tested when two or three months old, and it is found that less than 0.5 per cent. respond to the test, the others being free from tuberculosis. This shows that tuberculosis is rarely inherited. The milk from the reacting section is sent to the creamery. It is there separated, and the cream and skim-milk are pasteurized separately. At intervals of from six months to a year the healthy cattle are retested with tuberculin, and if reactions are obtained the reacting animals are transferred to the other section. Thus, gradually the tuberculous cattle are eliminated from the herd. As they develop physical signs of tuberculosis they are slaughtered, or they are fattened and sent to the slaughter-house, where they are killed under veterinary inspection. The healthy section is replenished partly by calves from tuberculous cows. In this way tuberculosis is being gradually eliminated in the herds treated according to this system.

More than 2000 herds have been treated in this way in Denmark, and the results are exceedingly encouraging. It is to be noted, however—and this is shown very clearly by Professor Bang's report—that the results are only good where great care is observed and where all of the precautions that are recommended are closely observed. That is, the Bang method could not be efficient if the separation of the reacting from the healthy cattle were not complete. It would not be efficient if the calves from the reacting cows were not fed as indicated and removed from their dams immediately after birth, nor would the Bang method be safe from the public health standpoint if the reacting cows, when killed by the butcher, were not killed under veterinary supervision and their carcasses carefully inspected. Further, the Bang method would not be safe, either as regards the farmer's cattle or the consumer of his products, if the cream and skim-milk from the tuberculous herd were not heated to a point that insures the destruction of the tubercle bacillus. Then, in addition, it is necessary that there should be two sets of attendants for these two lots of cattle. Or, if but one set, certain very necessary but arduous precautions as to shoes, clothing, washing the hands, etc., must always be observed. Without all of these details the Danish system cannot succeed. Therefore, before the Danish system is recommended for general use in this country, the conditions should be carefully considered, and it should be ascertained beyond peradventure that the requirements of the system can be met before it is recommended for general adoption. In other words, these questions must be answered: Can the American, or more particularly, the Pennsylvania farmer, dispose of milk that is known to come from tuberculous cattle? Is there a market for it? If not, the benefits of the system are very materially reduced. Then, if there is a market for such milk and the milk be heated to the necessary degree, thus making it safe for manufacture or consumption, can the necessary conditions as to separation and separate care be observed on the ordinary farm? Will the calves be removed immediately after they are born, and fed only on the milk from the non-reacting section or on the Pasteurized milk of cows from the reacting section? Will the reacting cows themselves, if any of them are fattened and sold for slaughter, be sold in such a way as to insure their slaughter under veterinary inspection; and then, how many farmers would care to deliberately and publicly maintain on their premises a number of cows that were known to be afflicted with tuberculosis? Last of all, we have another very pertinent question: Is it necessary in the United States, so far as the ordinary dairy cattle are concerned, for us to follow this method? In Denmark the percentage of tuberculosis is very high; more than a quarter of the cattle appear from the records to be tuberculous. In Pennsylvania it cannot be that the percentage is above 3 per cent., and probably not more than 2 per cent., so is it not cheaper in the end to at once dispose of the tuberculous animals? I do not mean to answer this question at this time or to offer an opinion upon the general advisability for us of the Bang method of suppressing tuberculosis, but I do wish to call attention to the difference in our conditions and to suggest several lines of inquiry that must be followed before the points necessary to the settlement of this matter are brought out.

Following this paper by Professor Bang, which I have gone into at some length, and have departed from very considerably in order to fully explain its import to us, there were a number of other papers of veterinary interest. One of these, prepared by Professor Nocard, and on the relation of avian to human tuberculosis, is, I believe, of very high importance. Professor Nocard has recently made a comparative study of the bacilli of human and avian tuberculosis. This study is of great importance at this time on account of the suggestions that have been made lately as to the possibility of great differences between bovine and human tuberculosis, and it has been inferred by some that these differences may be so great as to remove bovine tuberculosis from the domain of public health and place it completely and solely within the field of agriculture. Professor Nocard's work tends to unite the different forms of the tubercle bacillus more closely, and is in sharp contrast to the views that I have referred to. Professor Nocard has succeeded in transforming the bacilli of tuberculosis of birds into germs which respond to all of the tests for human tuberculosis to which he has subjected them. In other words, he does not claim that the germs of tuberculous birds and animals are merely very much alike, but he has shown that the germ of bird-tubercle can actually be changed into that of the other. This he has accomplished by a very ingenious method, the method of culture *in vivo* of Roux and Metchnikoff. This method consists in placing a culture of human tuberculosis in glycerin bouillon into a collodion sac, which is then inserted into the peritoneal cavity of a chicken. It is allowed to remain in this location for from six to eight months, the chicken is then killed, the sac removed, and it is found that the germ is changed so that it produces a form of tuberculosis in rabbits that is characteristic of the avian germ, and morphologically and culturally it corresponds to the same organism. Indeed, Professor Nocard goes so far as to claim that tuberculosis of fowls may be transmitted to man, and concludes by saying that the bacilli of avian and human tuberculosis are not two distinct species, but merely two varieties of the same species.

Attention was also called at the meeting to the recent reports of tuberculosis in fish, in which it appears that fish were infected by human sputum thrown into a pond, so that recent European investigations tend to further unify the various classes and varieties of tuberculosis. A great deal was said at the Congress about the new treatments for tuberculosis, of various forms of tuberculin, serums, antitoxin, etc. A number of claims were made for these remedies, but none of them was substantiated by sufficiently extensive reports to encourage us very much.

Perhaps more papers were read on the subject of sanitaria for consumptives than on any other. The world-wide movement for sanitaria for consumptives is most impressive. It appears that efforts are being made in all civilized countries for the isolation of the tuberculous, for the disinfection of the apartments occupied by them, etc.

Sanitaria are already provided for wealthy people in most countries, but there is little provision as yet for the poor. There can be little doubt, judging from the great interest that is now being taken in this subject and its immense importance, that sanitaria will in time be provided where the poor can be treated for consumption and other forms of tuberculosis, as well as these classes are now treated in hospitals for other ailments.

In the resolutions that were adopted by the Congress the various governments were requested to adopt some means for the prevention of the fraudulent use of tuberculin for the purpose of disguising the existence of tuberculosis of animals that were intended for sale or export. The following were also adopted : *Whereas*, in consideration of the incessant progress of tuberculosis of cattle, which constitutes a grave public loss and menaces public health, and that contagion is the only true cause of its progress, *be it resolved*, that there is urgent necessity for legislative measures in the following particulars : (*a*) The separation of the diseased animals from those that are healthy. (*b*) The prohibition of the sale of diseased animals for any purpose other than for slaughter. (*c*) The inspection of dairies devoted to the production of milk designed for sale to the public, and the immediate destruction of all cows suffering with tuberculosis of the udder. (*d*) The sterilization, or at least the Pasteurization, of milk used for the production of butter and cheese in quantities. (*e*) The generalization of the public service for the inspection of meats under a system analogous to the one that has been in operation in Belgium for a number of years.

The Congress made visits to several laboratories and hospitals, and was terminated by a banquet.

The interest that is being taken in the subject of tuberculosis is increasing everywhere. Information in reference to it is being disseminated, and a true understanding of the disease is beginning to prevail, so that it is altogether probable that progress in prophylaxis will be made rapidly and the death-rate from consumption will steadily fall.

While in Paris I had the pleasure of making several visits to the Veterinary College at Alfort, one of them in company with Dr. A. Liautard. Although it was vacation, the clinics were continuing and work was going on in some of the laboratories. I found that a great change had been made in the Alfort School during the past few years, and that a large building containing the laboratories, museum, dissecting-rooms, etc., had been erected. The old building, in which the dissecting-room was formerly located, is now used as a chemical laboratory. Dr. Liautard made arrangements for us to visit the stables of the Paris Omnibus Company. This company controls the omnibus and horse-car lines of Paris, and has 18,000 horses. Most of these horses are Percherons and Percheron grades, about one-third are stallions, one-third geldings, and one-third mares. In some stables the horses are all stallions, in others geldings, and in others mares. The stallions are usually worked in teams, three abreast, and do not give rise to any disturbance whatever. The superintendent told me that they bought all the stallions they could get, and that they would have all stallions if they could find them. It is claimed that stallions are not only better looking, but more spirited, stronger, and more durable than geldings or mares of the same breed. The stables are very well constructed, the horses are kept on two floors, and feed is stored in separate buildings. The floors are made of granite blocks covered in the stalls with eight inches of peat moss. This method of bedding has been practised for several years, and the company finds that it is very desirable to have the horses stand on the soft bed when not in service on the streets. They rest better, and their feet and legs wear longer with this arrangement. These 18,000 horses are fed American oats and American corn. Their daily ration consists of 2 pounds of

African beans, 8 pounds of shelled corn, 8 pounds of oats. In addition they receive 10 pounds of cut hay or oat-straw. The horses are required to go from twelve to sixteen miles a day, and last at this service a little over five years.

A number of automobiles were seen on the boulevards and parks of Paris, but, so far as I could ascertain, these were not used for any public service. They belong to individuals, and are kept for brief outings in the city and immediately surrounding country. They are subject to numerous mechanical objections, and were not used in anything like the numbers that I expected to see, judging from the accounts that have been printed in papers. Moreover, I was told that not as many are in use now as two years ago.

From Paris I went to Switzerland to ascertain the methods employed in that country in dealing with tuberculosis of cattle, and to visit the Veterinary School at Berne. There is not a great deal of tuberculosis among the cattle of Switzerland, and the plan adopted is a radical modification of the Danish system. Cattle are tested with tuberculin, and many of them are killed at once. The killing, however, is done in slaughter-houses where the carcasses are inspected by veterinarians, and if the flesh is fit for food it is placed upon the market, subject in some cases to certain restrictions. Otherwise, the carcass is condemned outright. If the animals are not ready for slaughter, and are in such a condition that they can be improved by feeding, they are kept alive, but are all marked officially by removing a triangular piece from one ear. This means that the animal has responded to the tuberculin-test, and prospective purchasers are thereby cautioned.

In Berne I had great pleasure in visiting the veterinary school. The little country of Switzerland supports two veterinary schools at public expense. Besides the one I visited there is another at Lucerne. The Berne school is quite old, but the buildings were destroyed by fire a few years ago, so that the equipment is entirely new. The buildings are both beautiful and substantial, well arranged, convenient, and commodious. They comprise a residence building for the principal professors, in which there are a few lecture-rooms, a building for pathology and anatomy, the hospital, postmortem building, and the farriery. I was received and entertained by Professors Hess, Guillebeau, and Noyer, who took great pains to show me the entire equipment and furnish me with the details of the administration. This institution (as, indeed, are all of the European veterinary colleges) is supported by the government, because there is a keen realization of the fact that it is to the advantage of the livestock interests and of the country to have available a force of well-trained men to look after the health of livestock and, indirectly, of the public, and to suppress and keep in check the various infectious diseases of animals that without control would render stock-breeding impossible.

In Germany I found great differences of opinion as to the measures which should be adopted by the country in dealing with the very important question of tuberculosis. The importance of tuberculosis of cattle is fully realized in Germany, but no systematic attempt has been made to suppress it because the distribution of the disease is so universal and the magnitude of the question is such as to appall the authorities. In Saxony more than one-quarter of the cattle killed for food are found to contain the lesions of tuberculosis, and in other districts the distribution of the disease is even greater. At present the government is expending a good deal of money in experiments with the Danish and other systems, and there seems to be no doubt that some general measures will be undertaken very soon.

In addition to the immense expense which the management of this question will involve, and which is, perhaps, the occasion for part of the delay, there is another factor which has contributed to defer action. That is, tuberculosis of cattle is hedged around by so many precautions in Germany that the disease has not the pressing public-health importance that it has in this country. I do not mean by this that the German is less apt to contract tuberculosis by eating the flesh or drinking the milk of a tuberculous cow than is his American cousin, but, in the first place, dangerous meat does not reach the market on account of the thorough systems of meat-inspection that are practised. Milk is always boiled, and the use of raw milk is almost as rare in Germany as the use of raw meat with us. So that even if the cattle were tuberculous to a much higher degree than they are, so long as these systems are in operation the public health is not seriously endangered. But while the government is casting around for a satisfactory means of suppressing tuberculosis, the disease in making strides, and it is generally recognized that it has gained great headway during the past few years.

In Berlin I found that the veterinary school had enlarged considerably since the time of my residence there. There is a new operating-room; a surgical clinic; Professor Dieckerhoff's clinic has been enlarged; there is a new stable for the dairy that is maintained on the premises; there are several new residences on the grounds, and an institute of hygiene is now being erected for the department of which Professor Ostertag is the head. I desired to visit the new veterinary school which is being erected in Hanover, which it is claimed will be the most perfectly equipped of any in the world, but my time did not permit this visit.

From Germany I went to Denmark, and visited the veterinary and agricultural college in Copenhagen. Professor Bang had made arrangements for my visit, and great pains were taken to show me everything that I desired to see. I am also under obligations to Mr. Haral Morkeberg, veterinarian, for his great kindness in showing me the various points of interest, such as laboratories, the milk-supply company of Copenhagen, dairy farms, etc.

The veterinary college in Copenhagen is exceedingly well equipped. The agricultural college is in connection with it. The course of instruction covers four years, and is very thorough. I shall not allude further to my visit to Denmark, as I have already commented on Danish conditions of agriculture, etc., in connection with the reference to the article read by Professor Bang in Paris.

My trip was a very hurried one, but I managed to learn something of the conditions in four countries—France, Germany, Switzerland, and Denmark—and the impression that I received was that the most active interest in being taken in tuberculosis and in the suppression of this disease in Europe, and, further, that interest in veterinary education is increasing and that the outlook for useful veterinary occupation is most promising.

